# Parental Reconstruction from a Half-Sib Population of Stoneless Jujube *Ziziphus jujuba* Mill. Based on Individual Specific SNP Markers Using Multiplex PCR

**DOI:** 10.3390/plants13223163

**Published:** 2024-11-11

**Authors:** Muhammad Tahir, Yue Ren, Bo Wu, Meiyu Li, Mohamed Refaiy, Ming Cao, Decang Kong, Xiaoming Pang

**Affiliations:** 1 State Key Laboratory of Tree Genetics and Breeding, National Engineering Research Center of Tree Breeding and Ecological Restoration, Key Laboratory of Genetics and Breeding in Forest Trees and Ornamental Plants, Ministry of Education, College of Biological Sciences and Biotechnology, Beijing Forestry University, Beijing 100083, China; malikuaf2011@gmail.com (M.T.); yueren9918@bjfu.edu.cn (Y.R.); wb219105@163.com (B.W.); meiyulily@163.com (M.L.); m_refaiy@bjfu.edu.cn (M.R.); 2 National Foundation for Improved Cultivars of Chinese Jujube, Cangzhou 061000, China; i2008caoming@126.com (M.C.); kongdecang@126.com (D.K.)

**Keywords:** stoneless fruit, multiplex PCR, SNP genotyping, parentage analysis, open pollination

## Abstract

The selection of unique and individual-specific SNPs is important as compared with universal SNPs for individual identification. Therefore, the main significance of this research is the selection of specific SNPs in male parent and the identification of offspring with these specific SNPs in their genome by multiplex PCR, which is utilized for genotyping of 332 half-sib plants of *Ziziphus jujuba*.This cost-effective method makes as much as possible to utilize the same amount of each pair of various targeted loci primers. After PCR amplification of targeted genome parts, the mixed products can be directly used in a next-generation sequencing platform. We concomitantly amplified 10 unique SNP loci at 10 different chromosomes of male JingZao 39 plants in 332 half-sib plants and sequenced them on the Illumina Novaseq 6000 platform. Analysis of SNP genotyping accuracy of 332 half-sib plants showed that all 10 unique SNPs in all 332 plants were correctly amplified in this multiplex PCR method. Furthermore, based on Mendelian inheritance, we identified 124 full-sib plants that have 10 unique SNPs in their genomes. These results were further confirmed by whole genome resequencing of 82 randomly selected half-sib plants, and the identity-by-descent values of all full-sib plants were between 0.4399 to 0.5652. This study displayed a cost-effective multiplex PCR method and proper identification of pollen parent through specific SNPs in half-sib progenies and firstly obtained a full-sib population between ‘Wuhezao’ and ‘JingZao 39’, segregating for stone and stoneless fruit.

## 1. Introduction

*Ziziphus Jujuba* Mill. is an economically important fresh fruit well known for its distinctive flavor and significant nutritional benefits [1]. Jujube fruit is one of the most consumed fruits in the Rhamnaceae family because of its positive effects on human health and specific flavor. Jujube fruit originated from China where it has been cultivated for more than 4000 years [2]. Jujube trees are deciduous and seasonally shed their leaves; flowering starts from April to June while fruit maturation takes place from August to October in China [3]. In horticultural fruits, such as grapes, apples, peaches, and jujube, somatic mutations remain stable during vegetative propagation [4]. Different DNA sequencing techniques based on molecular markers are currently utilized to identify these mutations. Among molecular markers, unique SNP markers are mostly utilized for varietal identification and parental tests [5]. Because of easy automation and data integration capabilities, unique and specific SNPs are perfect genetic markers for varietal identification and parentage analysis. The role of unique SNPs in the identification of bud varieties is centered on their potential to serve as specific genetic markers for distinguishing one bud variety from another. These SNPs are specific to particular varieties and are not commonly found in other varieties. Analyzing the presence or absence of these specific SNPs provides a unique genetic signature for each variety and parental identification [4,6]. In parentage analysis, next-generation sequencing (NGS) is rapidly gaining momentum. The earlier application of NGS was to utilize sequencing data to obtain microsatellite markers and SNP markers that can easily be amplified through PCR [7,8]. Different strategies are being integrated into NGS for verification of SNPs and different mutations by resequencing. These strategies include long PCR [9], hybrid capture [10], multiplex PCR [11], and molecular inversion probes [12].

With the accessibility of genomic resources and multiplexed methods to concurrently analyze multiple single nucleotide polymorphisms (SNP), scientists have approached these unique SNPs for paternity analysis and bud varieties identification [13,14]. SNP genotyping in more than 600 grape varieties has been utilized to check parental relationships and to recognize the important ancestors of Italian cultivars and well-related accessions [15]. In open-pollinated *eucalypts*, 106 SNP markers have been selected for successful reconstruction of pedigrees and SNP markers data perform better than microsatellite data [13]. A human tumor cell study has shown that patient-specific SNPs contribute to genetic heterogeneity, representing genetic changes that are not present in all common tumor cells; they are only present in specific tumor cells and are patient-specific SNPs [16]. Therefore, SNP markers are gaining popularity for the identification of individuals carrying specific SNPs. 

In fruit trees such as *Z. jujuba* Mill. which have very small flowers, the production of the half-sib families is the best option for progeny testing. Therefore, the use of individual SNPs is very useful for progeny testing and these SNPs are able to distinguish different male parents of somatic variation. Half-sib families are a natural way of creating genetic variations [17]. A study of genetic variations in half-sib families based on unique SNPs shows that half-sib populations behave differently for the traits under observation [18,19,20]. Although paternity analysis problems have been widely solved by microsatellite markers, some problems still exist. For example, microsatellites were successful in paternity studies in those species in which microsatellites were abundant and highly polymorphic in the genome, i.e., most fishes [21]. However, microsatellite markers are unable to differentiate budding varieties, making paternity testing difficult. In addition, microsatellites require a large initial investment in locus identification and designing locus-specific primers and optimization of PCR conditions [22]. So, for the differentiation of budding varieties with somatic variations in male parents, the use of individual-specific SNPs is very important.

As compared to traditional PCR, multiplex PCR is a suitable choice for genotyping many sets of SNPs, as it is time-saving, cost-effective, flexible to design, and, at the end of the reaction, a smaller amount of template DNA remains [23]. Multiplex PCR has the ability to amplify a large set of SNPs in a single round of PCR. Multiplex PCR combined with next-generation sequencing is a marker detection method that reduces costs and increases product efficiency [24]. The multiplex PCR approach has been widely used in vegetables, crops, and animals, but very little information about the application of multiplex PCR is available in fruit trees. 

In our study, we established a two-round multiplex PCR method in *Z. jujuba* Mill. fruit, followed by high-throughput sequencing that targeted 10 SNP markers specific to Z. jujuba (JingZao 39) male parents. Furthermore, we developed a novel method of parentage assignment through parental reconstruction by using panels of unique SNP markers and the utilization of these SNP markers of individual specificity in budding varieties differentiation in *Z. jujuba* Mill. because of their uniqueness. The results verified the application of multiplex PCR in fruit species and the power of SNPs in paternity tests. Our study provides a valuable reference for progeny identification, budding varieties differentiation, and molecular breeding processes. 

## 2. Results

### 2.1. Selection of JingZao 39 Male Trees for Parental Reconstruction of Half-Sib Population

Totally, 332 open pollinated seedlings from ‘Wuhezao’ were obtained and ‘JingZao 39’ was selected as the male tree in our study for parental reconstruction. The JingZao 39 genome was sequenced two times (JingZao 39A and JingZao 39B) so there was a minimum chance of selecting false SNPs; this gave a big reason to give priority to the JingZao 39 male tree among 150 other male trees for parental reconstruction. A unique name (JingZao 39A, JingZao 39B) was given each time the JingZao 39 genome was sequenced.

### 2.2. Selection of Individual Specific SNPs of JingZao 39

**Figure 1:**
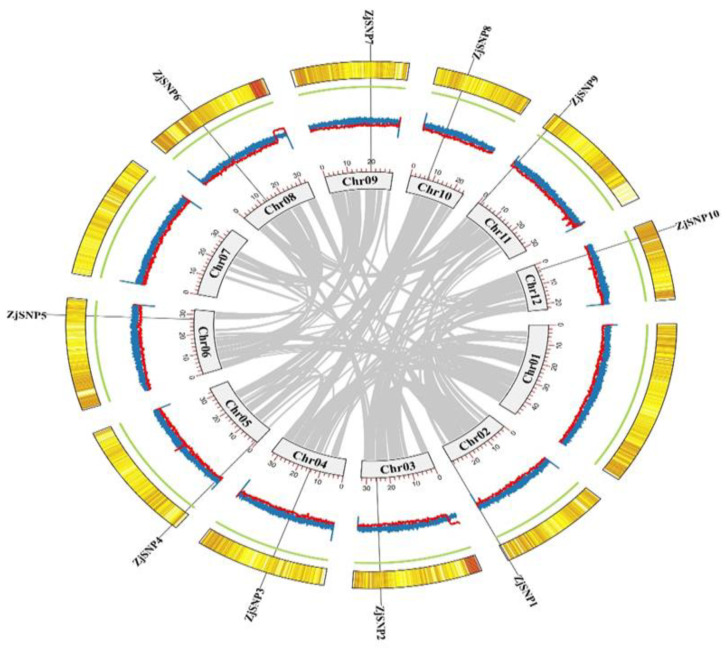
Circos plot of Z. jujuba Mill. (JingZao 39) genome assembly. Location of 10 SNPs loci on 10 different jujube chromosomes. Yellow color gene density heatmap, green color line N ratio, blue-red color GC skew.

Initially, we selected 80 SNPs from JingZao 39 genome sequenced data. Selected SNPs were randomly located on each chromosome. All 80 SNPs were amplified by using a PCR machine and PCR products were loaded on gel to observe a single sharp band. Each SNP gave a single sharp band and was selected and Sanger sequenced. A total of 50 SNPs gave single band and sequencing results, which were analyzed using BioEdit software https://bioedit.software.informer.com/7.2/. According to the aim of our study, 10 perfect SNPs were selected for designing multiplex PCR primers that were located on each chromosome of the JingZao 39 variety except chromosome 1 and chromosome 7, as shown in Figure 1. Finally, a primer mix group of 10 SNPs was composed for variety identification by target SNP sequencing. 

**Figure 2:**
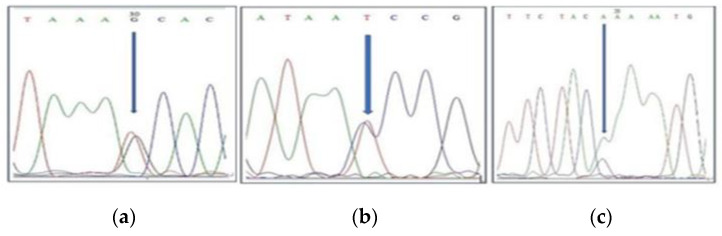
Heterologous peak at SNP position. (**a**) Chromosome 2; (**b**) chromosome 4; (**c**) chromosome 5.

Out of 80 SNPs that gave sharp bands on a gel image, only 10 SNPs showed clear and high heterologous peaks in BioEdit software. The only SNP that showed a heterologous peak at the SNP position was considered as a perfect SNP, as shown in Figure 2. 

### 2.3. Confirmation of Male Parents of Half-Sib Plants Using SNPs and Identity by the Descent Method

The purpose of the study was to find unknown male parents of half-sib plants whose female parent was known. For this, 10 well confirmed SNPs markers of male JingZao 39 variety were used for target sequencing. Genomes are formed in a pair of chromosomes carrying a copy of the male parent and a copy of the female parent; haplotype string (0/1) for half-sib plants samples at target SNP position in the VCF files are the offspring of unknow male parent JingZao 39. In 10 sets of SNPs, any half-sib plant samples that show haplotypes string (0/1) at 1 of 10 SNP-targeted positions is considered JingZao 39 offspring. On the base of the haplotype string (0/1), we identified 124 full-sib descendents of JingZao 39. To confirm the ancestor of these 124 half-sib plants, 82 randomly selected half-sib plants were used for whole-genome resequencing. The pairwise matrix of IBD values was used for the confirmation of the first-degree relationship (parent–offspring). The expected value of IBD for first-degree relationships is 0.5 [25]. However, obtained IBD values for parents–offsprings are expected to differ because of genotyping error, uneven SNP density, and errors in reference genome assembly. IBD value of 82 JingZao 39’s offspring were between 0.4399 and 0.5652 (Supplementary File S2) by mapping with both times sequenced genome data of JingZao 39A and JingZao 39 B. Pairs of samples with IBD values greater than or equal to 0.4358 and less than or equal to 0.5625 are considered as putative first-degree relatives. The IBD values of 82 selected hald-sib plants were the same in JingZao 39A and JingZao 39B. Whole genome resequencing data analysis based on IBD values further confirmed the results of SNP-based parent identification.

### 2.4. Two-Round PCR

**Figure 3:**
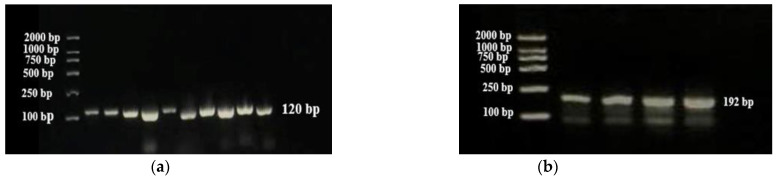
PCR amplification (**a**) PCR1 amplification of targeted 10 SNPs with multiplex PCR from 10 different samples, each band represents a product of 10 targeted SNPs loci with a final length product of 120 bp. (**b**) PCR2 amplification by using the PCR1 product as a template after adding adapters and index sequencing from 4 different samples with a final length product of 192 bp. Each band represents a product of 10 SNP positions.

We designed two rounds of multiplex PCR to obtain the maximum uniformity of the amplicons. In the first round of PCR, low concentrations of SNP target primers containing i5 and i7 adapters gave perfect bands without primer dimerization. SNP primers annealed perfectly with target regions and SNP-specific regions were amplified without making dimerization, as shown in Figure 3a. In the second round of PCR, barcodes and index sequences were used to amplify the target region of each sample. Different target loci of each sample were performed in a single tube and all loci were perfectly amplified after adding barcodes and an index with primers (Figure 3b). Different barcodes were given to different samples to avoid mixing of samples after making pools of all samples in single tubes for sequencing. These barcodes gave a unique identity to each sample.

#### Multiplex PCR Data Description

**Figure 4:**
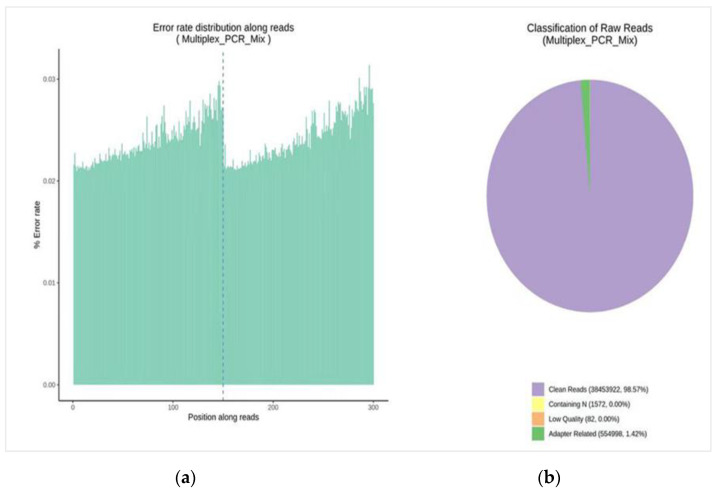
(**a**) Error rate distribution along read position. (**b**) Classification of raw reads.

The data set described in this article is targeted SNP paired-end sequencing results of the NovaSeq 6000 platform. The sequencing quality score of the data set falls between Q30 and Q40, where Q30 shows 99.9% correct base recognition and Q40 shows 99.99% correct base recognition. The horizontal line indicates the base position of the read and the vertical line indicates the single base mass value. The first half is the mass distribution of the first-end sequencing reads of the two-ended sequencing, and the second half is the mass distribution of the sequencing reads at the other end. The error rate of a single base along the position of the read is under 0.03% (Figure 4a).

**Figure 5:**
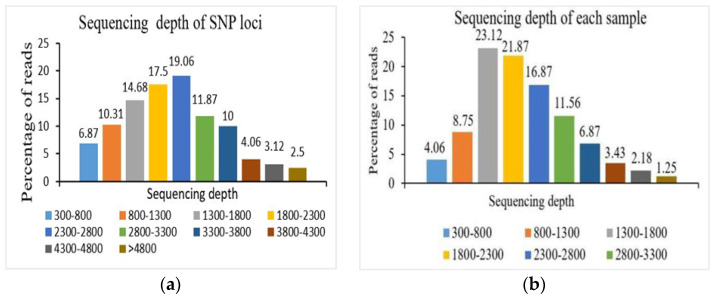
(**a**) Sequencing depth of 10 perfect SNPs, (**b**) sequencing depth of each DNA sample.

The total GC content of the data was 41.42%. We obtained 39,010,574 raw reads; out of these, 38,453,922 were clean reads, which is 98.57% of the total reads followed by 1.42% of the adapter-related sequence and 0.00% of reads with N base sequences (Figure 4b). Sequencing the depth of each SNP for each sample was calculated and the sequencing depth of 93.13% of reads of SNPs was more than 800×. The average sequencing depth of each SNP for all samples was 2300× (Figure 5a). The sequencing depth of 85.25% of reads of each variety was more than 1300× while the average sequencing depth of 320 DNA samples was 2000× (Figure 5b).

## 3. Discussion

### 3.1. Development of a Two-Round Multiplex PCR Assay

The development of successful multiplex PCR assays for both plants and animals becomes harder with the increase in multiplexing levels due to more chances of irregular primer–primer interactions [26]. Our multiplex PCR method utilizes the principles of the i5 and i7 adapter methods for Illumina sequencing and two rounds of PCR amplification in jujube fruit. Our results of two-round of PCR produced an average read sequence and our results of two rounds of PCR amplification were similar to other researchers who utilized two-round multiplex PCR techniques in *Triticum aestivum* L. [27]. The production of more average read sequences was likely because of two reasons: (1) the competition between primer pairs to utilize template DNA was similar and (2) the formation of primer dimmers was minimized. In our methods, targeted primers in PCR1 were completely utilized as there was a minimum primer dimerization. As there was no PCR purification step after PCR1, complete utilization of PCR1 primers tagged with i5 and i7 adapters is compulsory to avoid adapter dimers in PCR2. The utilization of the adaptor sequence in PCR1 not only reduced the time but also reduced the cost as compared to the three round multiplex PCR method in which adaptor primers were added in a third round of multiplex PCR [28]. The utilization of i5 and i7 adaptors for sequencing in our method is similar to other research, which utilized i5 and i7 sequencing adaptors [29]. These generic nucleotide sequence sites decrease amplification biases among different targeted amplicons by normalizing primer hybridization kinetics [30]. This contributes high uniformity of targeted sequences amplification in the second round of multiplex PCR and as a result, an equal yield of PCR product is obtained for every targeted locus in the multiplex reaction. In the second amplification stage, i5 and i7 adapters tagged with different barcodes and index sequences uniformly amplify the first round PCR product without any discrimination of targeted DNA. In multiplex PCR, a uniform PCR product of different targeted DNA sequences is an important parameter [31]. In our two-round multiplex PCR method, we amplified 10 SNPs from 332 DNA samples of jujube fruit and a sequencing depth of 85.25% reads of each variety was more than 1300×; similar results of two-round PCR to simultaneously amplify different SNPs have been studied in *Triticum aestivum* L. [27]. These results showed that our two-round multiplex PCR uniformly amplified different SNPs in jujube, similar to other multiplex PCR assays including High-plex PCR, multiplex PCR with MTA-seq, and three-round multiplex PCR [23,32,33].

### 3.2. Multiplex PCR-Based Target SNP-Seq Technology

With the passage of time, many new ways for SNP genotyping have been reported, and the use of SNP technology has sped up genetic research [34]. However, more labor costs and poor efficiency are nevertheless challenging situations for modern SNP genotyping platforms [35]. Currently, NGS technology based on a few SNP genotyping methods has begun to boost up methods, such as GBTS, Ampseq, and Seq-SNP, but these methods have some drawbacks, such as SNP amplification primers needing probe hybridization. Multiplex PCR-based target SNP-seq technology in fruit crops is not studied well because of difficulties connected with assay development [36]. Both traditional PCR and multiplex PCR are based on the same fundamental principle of polymerase chain reaction, where DNA is amplified through repeated cycles of denaturation, annealing, and extension. The primary difference lies in the number of target sequences amplified. Traditional PCR amplifies a single target, whereas multiplex PCR amplifies multiple targets simultaneously in a single reaction [37]. The use of new approaches for multiplex PCR assay development in fruit crops has the potential to speed up fruit crop genotyping research. In our study, we developed target SNP-seq-based genotyping methods and used them for a genotyping study of 332 *Z. jujuba* Mill. plants. The multiplex PCR products with 10 perfect SNP amplicons were used for sequencing on the Illumina NovaSeq 6000 platform. Millions of reads obtained from the sequencing platform were used for alignment with the JingZao 39 reference genome in order to get perfect SNP genotypes. High sequencing depth of multiplex PCR in target SNP-seq methods enables high call rate scores and high precision of crop genotyping [38]. So, high sequencing depth makes it easy to catch the perfect sequence in the genome and the correct location in bioinformatics analysis, which enables us to decrease false positives and false negatives. In this study, the average read depth of the selected perfect SNPs was more than 2300× (Figure 5a). Targeted SNP-seq technology had a clear edge in the amplification of hundreds of SNPs in comparison with traditional SNP methods and the complete process needs just only 3 days and USD 0.5 for every DNA sample. So, our method is a cost-effective, economic, and time-saving method to amplify many samples with more SNPs.

### 3.3. SNPs and Whole Genomes Resequencing-Based Paternity Analysis

Sexual reproduction in the *Z*. *jujuba* Mill. tree produces recombinant offspring through natural cross-pollination. The production of interested recombinant progenies for reasonable selection from highly interested parents with artificial cross-pollination is a challenging target in *Z*. *jujuba* as well as for other fruit trees because of its tiny flower size [39]. However, random cross-pollination concerning fertile parents in fields and poly cross blocks results in high-fruit settings. Thus, open cross-pollination is a cost-saving and easy way to generate many seedlings for better genetic selection [40]. This study also evaluated parentage analysis by using heterozygous half-sib progenies produced from open cross-pollination. Natural cross-pollination in *Z*. *jujuba* generated half-sib families with a wide range of heterozygosity. Molecular markers, especially SNP markers, are being widely used for parental reconstruction in half-sib families originating from open cross-pollination. Individual specific SNPs can be utilized in parentage identification in plants, especially in situations where these rare variants are stable, heritable, and unique to specific individuals. Parental reconstruction analysis of half-sib families takes the advantage that we have earlier information that certain groups of half-sib offspring originated from common maternal parents [41]. Different kinds of genetic markers that are firmly transmitted to offspring are utilized for parentage analysis. Offspring share one allele from each parent, so known unique SNP alleles can be easily traced in the half-sib family. In our study on the base of unique and JingZao 39-specific SNP markers, we successfully traced the male parent (JingZao 39) of 124 out of 332 half-sib plants by observing the SNP calling the VCF file with a haplotype string (0/1). All half-sib plants that did not show haplotype string (0/1) at any target SNP location in the 10 Chromosomes set were considered as offspring of other male parents planted around the female parents. According to Mendelian inheritance, parents and offspring share one allele at a given locus for the co-dominant marker in diploid organisms [41]. So, JingZao 39 male parent’s offspring must contain one allele with a JingZao 39 specific SNP marker shared from the JingZao 39 male parent. Many other researchers also used SNP marker in the parental reconstruction of different open-pollinated plant species [13,15,42] but the main disadvantage of parent identification through IBD is that it needs whole genome resequencing, which is not economical. The utilization of these unique SNPs is not only in parental identification but also in human tumor cell identification as well as in bud mutation varieties. Our method is applied to half-sib descendants; in tumor cells, the identification of rare SNPs is utilized to identify genetic heterogeneity between different tumor cells and these rare SNPs are present in only specific tumor cells due to somatic variations [43].

We also used pairwise identity-by-descent (IBD) by using Plink software https://www.cog-genomics.org/plink2. to mark parent–offspring relationships by whole genome resequencing of 82 randomly selected full-sib plants identified as JingZao 39 offspring through the SNP marker. IBD spots the common segment of DNA inherited from common parents and is broadly used to characterize and analyze the genome. So, in parental reconstruction, the two offspring of common male parents share identical parts of the genome [44]. In diploid organisms, at any specific location in the genome, one of two homologous copies of DNA is transferred in the gamete for offspring. As meiosis is independent, each offspring’s independent random choices occur during gamete formation independently in the two parents of an individual. A single piece of DNA transferred in the gametes for all offspring of common ancestral is used in the identification of ancestors by IBD analysis as all individuals share a common piece of DNA [45,46,47]. IBD values are used to denote the offspring of a common ancestor. IBD values in our study for all JingZao 39 offspring were 0.4399 to 0.5652. In the parental reconstruction of the apple genome study, IBD values for all individuals in first-degree relationships were from 0.4158 to 0.5625 [25]. In the parental study of Italian grapevines, two methods including the count of Mendelian errors and identity by descent coefficient measured by Plink software were used for ancestoral identification [15] but this method of parental identification, compared to our method, finds it difficult to distinguish bud changes because it does not utilize somatic rare markers. However, the IBD analysis method can certainly distinguish and predict more complex genetic relationships. The advantage of our method to identify parent identification is that it does not need resequencing of the genome as it requires the IBD method, so it is more economical, and parental tests can be performed in a short time as compared to IBD. The SNP marker set we used in our study resulted successfully in parentage inference in *Z. jujuba* Mill. and the validation of results of SNP with IBD methods showed that our method perfectly identified the male parent of the half-sib family as a male parent identified by IBD. The significance of our method is that it is cost-effective and time-saving and does not require resequencing of the genome. The total costs for the sequencing of targeted SNP after multiplex PCR were just 172 USD, and per sample, the cost was 0.5 USD.

## 4. Materials and Methods

### 4.1. Selection of Genome-Wide Perfect SNPs in Z. jujuba (JingZao 39)

**Figure 6:**
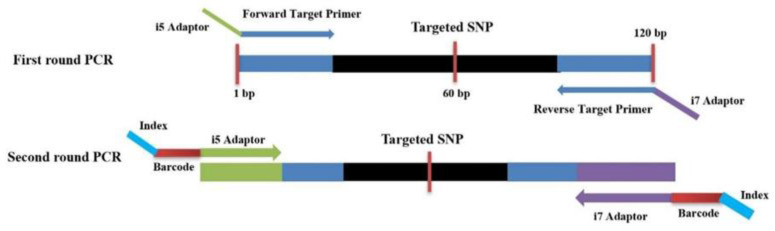
Diagrammatic overview of primer design. The first round of PCR contained specific primers attached to i5 adapters and i7 adapters, and the second round of PCR contained i5 adapters, barcodes, and index sequences.

The half-sib population was generated from a stoneless ‘Wuhezao’ tree surrounded by 150 pollen parents. As JingZao 39 male parent trees were located in close proximity to the stoneless mother tree, therefore JingZao 39 variety was selected for parental reconstruction. JingZao 39 is an independent bud variety containing unique SNPs specific to it. JingZao 39 fruits are larger in size and less likely to crack and are considered a better male parent [48]. The sequenced genome of JingZao 39 available at the NCBI under the accession number PRJNA979943 was used [49]. GATK’s HaplotypeCaller was used to create a VCF file containing JingZao 39 specific SNPs. First, specific mutation sites (0/1) contained in the JingZao 39 sample (JingZao 39A and JingZao 39B) were screened while other samples containing (0/0 or 1/1) did not belong to JingZao 39 (Supplementary File S3). Initially, 80 SNPs were randomly selected from 12 chromosomes of the JingZao 39 variety; primers were designed in such a way that the target region was at 60 bp with a total length of PCR product 120 bp, as shown in (Figure 6). These 80 SNPs were amplified by using DNA extracted from male JingZao 39 and sent for Sanger sequencing. The results of Sanger sequencing were analyzed with BioEdit software for the identification of SNPs. Out of 80 SNPs, 10 unique and JingZao 39 specific SNPs (highlighted with a red line in Supplementary File S3) that showed sharp single bands on the gel imaging system and gave high peaks in BioEdit software were selected from 10 different chromosomes in such a way that one unique SNP was from each chromosome except chromosome 1 and 7 and it was hypothesized that all offspring of JingZao 39 will contain a 0/1 mutation site while other jujube varieties will not contain a 0/1 mutation site at these 10 SNPs positions. Different primers were designed to amplify these SNP positions with a final length of 120 bp. The locations of these 10 perfect SNPs on 10 different chromosomes of the JingZao 39 variety and primers are given in Table 1

### 4.2. Primer Design for Multiplex PCR

To obtain target PCR products, we designed two rounds of multiplex PCR with target sequences and adaptor sequences. More briefly, in the first round of PCR, target sequences of SNPs were amplified from DNA extracted separately from the leaves of 332 half-sib plants by using the Plant DNA Kit (Vazyme, Nanjing, China) according to the manufacturer’s instructions, while the purpose of the second round of PCR was to differentiate DNA samples on the base of unique barcode sequences. Primers are designed in such a way that each forward primer contains an i5 adaptor and each reverse primer contains an i7 adaptor. The primers used in the first round of multiplex PCR are given in Table 2 while primers used in the second round of multiplex PCR are given in Table 3. In the first round of PCR, universal primer i5 adaptors for Illumina sequencing were added with targeted forward primers, while universal primer i7 adaptors for Illumina sequencing were added with targeted reverse primers, as shown in Figure 6. 

A total volume of 40 µL was used in the first round of PCR containing 17 µL mixed primers with a final concentration of each primer of 0.05 μM, 3 µL of template DNA (35 ng/µL) extracted from leaves of half-sib plants, and 20 µL of 2X Rapid Taq Master mix enzyme. Each PCR tube contained DNA from different half-sib plants. The thermal cycling conditions of the PCR machines were as follows: 95 °C for 10 min, 35 cycles (95 °C for 20 s, 56 °C for 2 min, 75 °C for 15 s) and a final extension at 72 °C for 5 min. After completion of the first round of PCR, 2 µL of the first round of PCR product was used as a template for the second round of PCR. A total volume of 20 µL was used in the second round of PCR containing 2 µL of the first round PCR as a template, 1 µL forward primer (i5 adaptor) containing a unique barcode and index sequence, 1 µL reverse primer (i7 adaptor) also containing a unique barcode and index sequence (Figure 6), 10 µL of 2X Rapid Taq Master mix enzyme, and 6 µL distilled water. The thermal cycle conditions of the PCR machines were as follows: 95 °C for 3 min, 35 cycles (95 °C for 20 s, 55 °C for 20 s, 75 °C for 15 s), and final extension at 72 °C for 5 min. After completion of the second round of PCR, 10 µL from each sample was mixed together and sent to Beijing Novogene Bioinformatics Technology Co., Ltd., Beijing, China, for PCR-free library preparation and sequencing. 

### 4.3. Construction of the Target SNP-Seq PCR Free Library

A total amount of 1 μg DNA per sample was used as input material for the DNA sample preparations. Sequencing of PCR-free libraries was conducted and generated using the NEB Next^®^ Ultra™ DNA Library Prep Kit for Illumina (NEB, Ipswich, USA) following the manufacturer’s recommendations; index codes were added to attribute sequences to each sample. The library preparations were sequenced on an Illumina NovaSeq 6000 platform and paired-end reads were generated. Quality control is an essential step to guarantee meaningful downstream analysis. We used Fastp (version 0.19.7) to perform basic statistics on the quality of the raw reads.

### 4.4. Data Analysis

After quality control, the final clean reads had four parts: an index sequence, barcodes, an adaptor, and a target sequence. Multiplex clean data were first demultiplexed based on the barcodes of each sample with Cutadapt software https://cutadapt.readthedocs.io/en/v3.4/ [50] using demultiplexing paired-end reads with combinatorial parameters. After demultiplexing, adapter sequences were trimmed out with cutadapt software to generate SNP target sequences.

A new manual reference genome FASTA file of 2000 bp containing 200 bp from each chromosome carrying unique SNP was developed from the JingZao 39 reference genome big data file for alignment. The purpose of making a new manual reference genome was to do a quick alignment process. Borrow wheel aligner (BWA) and Samtools were used to align target SNP sequences with the new manually developed reference genome to generate SAM files [51]. SAM files were transferred into bam files using Samtools https://www.htslib.org/. The GATK HaplotypeCaller parameter was used to call the SNP variant from aligned bam files [52].

### 4.5. Parentage Analysis

First, a paternity test was analyzed on the base VCF files generated by the GATK HaplotypeCaller parameter. For this, the genotype of each sample was viewed for carrying homozygous or heterozygous alleles at specific SNP positions. Second, 82 half-sib offspring out of 124 whose male parent JingZao 39 was confirmed by viewing a heterozygous genotype (0/1) in the VCF file (Supplementary File S1) were used for whole-genome resequencing and all re-sequenced data of 82 half-sib plants are available at NCBI under accession number PRJNA979913 and Supplementary File S3. Data were analyzed to calculate pairwise identity-by-descent (IBD) using PLINK software [53]. 

## 5. Conclusions

We have designed a new multiplex PCR method for jujube fruit crops with small open-pollinated flowers combined with NGS for SNP genotyping. This method is cost effective, has good uniformity, and simultaneously detects many samples in an efficient way. Unique and individual specific SNP-based paternity analysis of *Z. jujuba* has been provided for the first time. Unique and individual specific SNPs can be used for the identification of horticulture, forest trees, and bud mutation varieties as compared to universal SNPs that are common to most individual in a population. 

## Figures and Tables

**Table 1:** Ten perfect and unique SNPs, their location on chromosomes, and their sequence.

Chromosome Number	SNP Position	Forward Primer	Reverse Primer
CHR2	29727994	TTGATGCATTATGAGAATAAGG	CAGTGTTTCCACATGATTCACAG
CHR6	27699126	TTATAAGAATGCATGGCAGGTA	TCGGTGTTGGTCAACCAGGCTCA
CHR4	14484283	TCAAGATGCACCAATTCTAAAGC	GGATCGGTCACCAAACGAGTCTC
CHR3	25613213	ACCAACAAAGGACATACCAAAG	TTAAACCCTATAAGTGATATG
CHR11	1081719	GATGCTTTGTCATTGATTTATGCTC	TTAAGACAACCACAACAAAATTTGC
CHR12	3218647	AGAACCCTGTACAACATGGAAG	CCGCAAGGTTCTCCTCTGAAT
CHR10	7524906	AAGAGGAGCTAGCTGGTACCTCC	GAGCTAACATTCAAGTGAAAAC
CHR9	19998728	AGCATACCTCAAACCCTCGGA	CATGAAAATTGAAAGGGAAGAAAG
CHR8	12945656	TCTAATCACATGTTGGTTAGG	ATAAGCCTTTATGATACAAACC
CHR5	992412	GTGCCCCTTGCATTTTAGTCTG	GCCATCTCAAGGAGTATAACTTTG

**Table 2:** Primers used in the first round of PCR; bold sequences represent i5 and i7 adapters.

Forward Primer PCR1	Reverse Primer PCR1
TCGTCGGCAGCGTCAGATGTTGATGCATTATGAGAATAAGG	TCTCACACATATTCTCTGTCCAGTGTTTCCACATGATTCACAG
TCGTCGGCAGCGTCAGATGTTATAAGAATGCATGGCAGGTA	TCTCACACATATTCTCTGTTCGGTGTTGGTCAACCAGGCTCA
TCGTCGGCAGCGTCAGATGTCAAGATGCACCAATTCTAAAGC	TCTCACACATATTCTCTGTGGATCGGTCACCAAACGAGTCTC
TCGTCGGCAGCGTCAGATGACCAACAAAGGACATACCAAAG	TCTCACACATATTCTCTGTTTAAACCCTATAAGTGATATG
TCGTCGGCAGCGTCAGATGGATGCTTTGTCATTGATTTATGCTC	TCTCACACATATTCTCTGTTTAAGACAACCACAACAAAATTTGC
TCGTCGGCAGCGTCAGATGAGAACCCTGTACAACATGGAAG	TCTCACACATATTCTCTGTCCGCAAGGTTCTCCTCTGAAT
TCGTCGGCAGCGTCAGATGAAGAGGAGCTAGCTGGTACCTCC	TCTCACACATATTCTCTGTGAGCTAACATTCAAGTGAAAAC
TCGTCGGCAGCGTCAGATGAGCATACCTCAAACCCTCGGA	TCTCACACATATTCTCTGTCATGAAAATTGAAAGGGAAGAAAG
TCGTCGGCAGCGTCAGATGTCTAATCACATGTTGGTTAGG	TCTCACACATATTCTCTGTATAAGCCTTTATGATACAAACC
TCGTCGGCAGCGTCAGATGGTGCCCCTTGCATTTTAGTCTG	TCTCACACATATTCTCTGTGCCATCTCAAGGAGTATAACTTTG

**Table 3:** Primers used in the second round of PCR; bold sequences represent barcodes; Italic sequences are index sequences.

Forward Primer PCR2	Reverse Primer PCR2
*AATAT***AGGTA**TCGTCGGCAGC	*CCTCT***GGAGA**TCTCACACATA
*AATAT***ATGAA**TCGTCGGCAGC	*CCTCT***CAAGA**TCTCACACATA
*AATAT***TACCA**TCGTCGGCAGC	*CCTCT***TACCA**TCTCACACATA
*AATAT***CAGAA**TCGTCGGCAGC	*CCTCT***CAGAA**TCTCACACATA
*AATAT***CAAGT**TCGTCGGCAGC	*CCTCT***CAAGT**TCTCACACATA
*AATAT***TTCGT**TCGTCGGCAGC	*CCTCT***TTCGT**TCTCACACATA
*AATAT***TCCGA**TCGTCGGCAGC	*CCTCT***TCCGA**TCTCACACATA
*AATAT***TGAGC**TCGTCGGCAGC	*CCTCT***TGAGC**TCTCACACATA
*AATAT***CTGAC**TCGTCGGCAGC	*CCTCT***CTGAC**TCTCACACATA
*AATAT***CCTCG**TCGTCGGCAGC	*CCTCT***CCTCG**TCTCACACATA
*AATAT***AGGTG**TCGTCGGCAGC	*CCTCT***AGGTG**TCTCACACATA
*AATAT***TCTAA**TCGTCGGCAGC	*CCTCT***TCTAA**TCTCACACATA
*AATAT***TTGGA**TCGTCGGCAGC	*CCTCT***TTGGA**TCTCACACATA
*AATAT***TCTAG**TCGTCGGCAGC	*CCTCT***TCTAG**TCTCACACATA
*AATAT***TCTGG**TCGTCGGCAGC	*CCTCT***TCTGG**TCTCACACATA
*AATAT***CTATT**TCGTCGGCAGC	*CCTCT***CTATT**TCTCACACATA
*AATAT***AGGCA**TCGTCGGCAGC	*CCTCT***AGGCA**TCTCACACATA
*AATAT***TTAGT**TCGTCGGCAGC	*CCTCT***TTAGT**TCTCACACATA
*AATAT***ATCCG**TCGTCGGCAGC	*CCTCT***ATCCG**TCTCACACATA

## Data Availability

All re-sequenced data of 82 half-sib plants are available at NCBI under accession number PRJNA979913. The sequenced genome of JingZao 39 available at the NCBI under the accession number PRJNA979943.

## Supplementary Materials

The following supporting information can be downloaded at https://www.mdpi.com/article/10.3390/plants13223163/s1: File S1. VCF file containing heterozygous 0/1 genotype. File S2. Identity by descent values. File S3. JingZao 39 SNPs and re-sequenced samples SNPs
